# Supplementary material for: Identification of photoperiod-induced specific miRNAs in the adrenal glands of Sunite sheep (Ovis aries)
Source: Front Vet Sci. 2022 Jul 22;9:888207. doi: 10.3389/fvets.2022.888207 (PMC9354845; doi:10.3389/fvets.2022.888207)
Supplement: Supplementary file 1 [file Data_Sheet_1.ZIP › Supplementary file/miRDeep2 file.zip!/miRDeep2 file/Know_Structure/LP42A3/oar-mir-127.pdf]

miRBase precursor : oar-mir-127
Total read count : 149763
oar-miR-127 read count : 146066
remaining reads : 3697

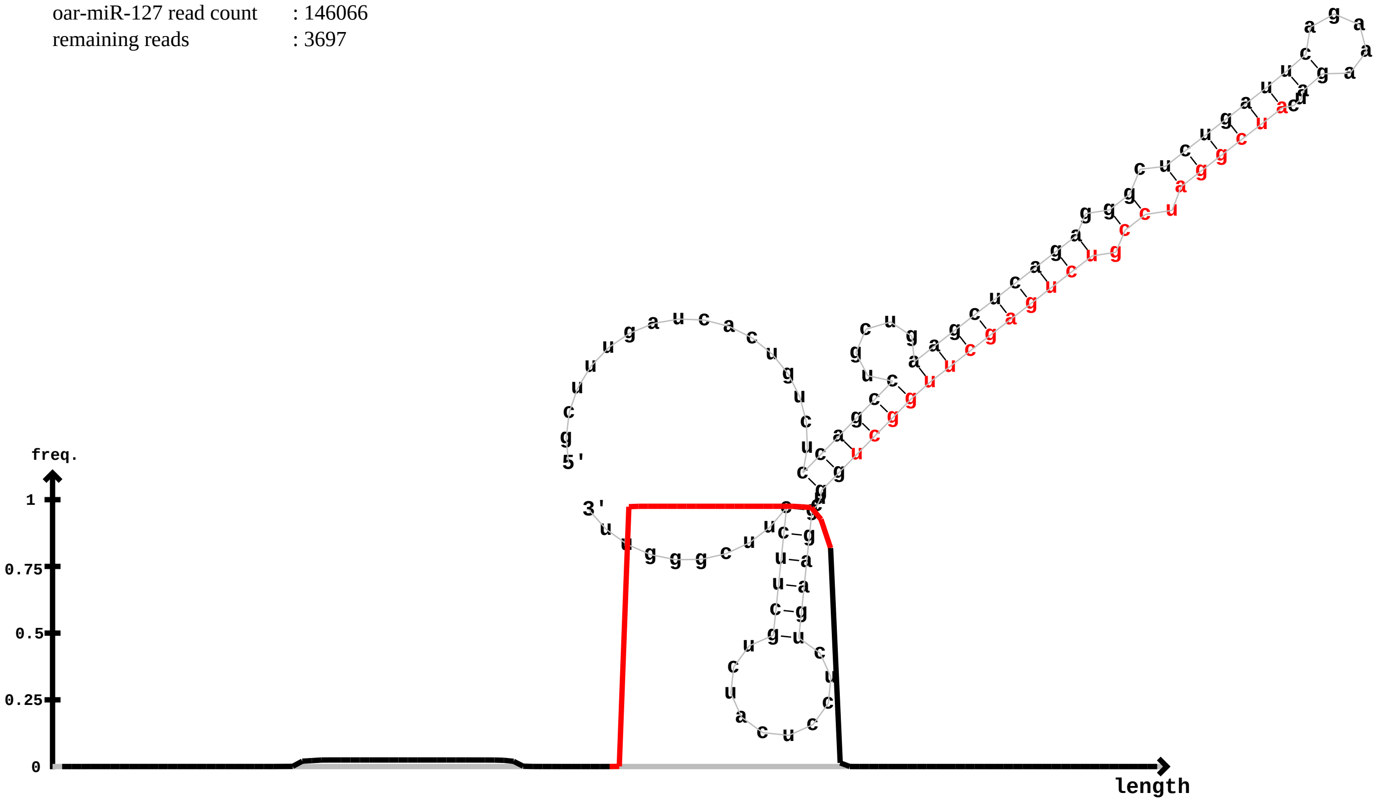

oar-miR-127

5'- gcuuugaucacugucuccagccugcugaagcucagagggcucugauucagaaagaucaucggauccgucugagcuuggcuggucggaagucuccucaucugcuuccuucggguu -3'	exp		
................((((((.....(((((((((.((.((((((((.....))..)))))).)).)))))))))))))))..((((((..........))))))........	reads	mm	sample
gcuuugaucacugucuccagccug..	1	0	seq
.cuuugaucacugucuccagccug..	6	0	seq
..uuugaucacugucuccagcc..	1	0	seq
..uuugaucacugucuccagccug..	3	0	seq
....ugaucacugucuccagccu...	1	0	seq
....ugaucacugucuccagccug..	4	0	seq
......................ugcugaagcucagagggcu...	1	0	seq
......................ugcugaagcucagagggcuc..	1	0	seq
......................ugcugaagcucagagggcucu...	3	0	seq
......................ugcugaagcucagagggcucug..	6	0	seq
......................ugcugaagcucagagggcucuga...	27	0	seq
......................ugcugaagcucagagggcucugau..	9	0	seq
......................ugcugaagcucagagggcucugauu...	4	0	seq
.......................gcugaagcucagagggcucug..	1	0	seq
.......................gcugaagcucagagggcucuga...	10	0	seq
.......................gcugaagcucagagggcucugau..	7	0	seq
.......................gcugaagcucagagggcucugauu...	1	0	seq
.......................gcugaagcucagagggcucugauuc..	1	0	seq
........................cugaagcucagagggcuU..	1	1	seq
........................cugaagcucagagggcuc..	3	0	seq
........................cugaagcucagagggcucC...	1	1	seq
........................cugaagcucagagggcucu...	5	0	seq
........................cugaagcucagagggcucug..	19	0	seq
........................cugaagcucagagggcucuga...	110	0	seq
........................cugaagcucagagggcucugau..	536	0	seq
........................cugaagcucagagggcucugauu...	2209	0	seq
........................cugaagcucagagggcucugauuc..	40	0	seq
........................cugaagcucagagggcucugauucagaa..	2	0	seq
........................cugaagcucagagggcucugauucagaaagauc...	1	0	seq
.........................ugaagcucagagggcucug..	5	0	seq
.........................ugaagcucagagggcucugau..	9	0	seq
.........................ugaagcucagagggcucugauu...	304	0	seq
.........................ugaagcucagagggcucugauuc..	8	0	seq
.........................ugaagcucagagggcucugauuca...	2	0	seq
.........................ugaagcucagagggcucugauucaga...	1	0	seq

oar-miR-127

```
gcuuugaucacugucuccagccugcugaagcucagagggcucugauucagaaagaucaucggauccgucugagcuuggcuggucggaagucuccucaucugcuuccuucggguu
..........................gaagcucagagggcucug......................................................................   1       0   seq
..........................gaagcucagagggcucugau....................................................................   3       0   seq
..........................gaagcucagagggcucugauu...................................................................   205     0   seq
..........................gaagcucagagggcucugauuc..................................................................   115     0   seq
..........................gaagcucagagggcucugauuca.................................................................   9       0   seq
..........................gaagcucagagggcucugauucaga...............................................................   1       0   seq
...........................aagcucagagggcucugau....................................................................   1       0   seq
...........................aagcucagagggcucugauu...................................................................   8       0   seq
...........................aagcucagagggcucugauuc..................................................................   2       0   seq
...........................aagcucagagggcucugauucag................................................................   5       0   seq
...........................aagcucagagggcucugauucaga...............................................................   3       0   seq
...............................................cagaaagaucaucggauccgucugagcuu......................................   1       0   seq
.......................................................ucaucggauccgucugagcuugg....................................   1       0   seq
.......................................................ucaucggauccgucugagcuuggc...................................   1       0   seq
........................................................caucggauccgucugagcuugg....................................   2       0   seq
........................................................caucggauccgucugagcuuggc...................................   3       0   seq
........................................................caucggauccgucugagcuuggcu..................................   1       0   seq
.........................................................aucggauccgucugagcuugg....................................   11      0   seq
.........................................................aucggauccgucugagcuuggc...................................   54      0   seq
.........................................................aucggauccgucugagcuuggcu..................................   9       0   seq
.........................................................aucggauccgucugagcuuggcug.................................   1       0   seq
..........................................................ucggauccgucugagcu.......................................   5       0   seq
..........................................................ucggauccgucugagcuu......................................   292     0   seq
..........................................................ucggauccgucugagcuug.....................................   441     0   seq
..........................................................ucggauccgucugagcuugg....................................   6560    0   seq
..........................................................ucggauccgucugagcuuggc...................................   16054   0   seq
..........................................................ucggauccgucugagcuuggcu..................................   120356  0   seq
..........................................................ucggauccgucugagcuuggcug.................................   2004    0   seq
..........................................................ucggauccgucugagcuuggcugg................................   20      0   seq
..........................................................ucggauccgucugagcuuggcuggu...............................   1       0   seq
...........................................................cggauccgucugagcuugg....................................   11      0   seq
...........................................................cggauccgucugagcuuggc...................................   31      0   seq
...........................................................cggauccgucugagcuuggcu..................................   175     0   seq
...........................................................cggauccgucugagcuuggcug.................................   21      0   seq
...........................................................cggauccgucugagcuuggcugg................................   1       0   seq
............................................................ggauccgucugagcuuggcu..................................   8       0   seq
............................................................ggauccgucugagcuuggcug.................................   2       0   seq
..............................................................auccgucugagcuuggc...................................   1       0   seq
```
